# Frequency and Risk Factors of Reproductive Coercion Among Pregnant Individuals: A Cross-Sectional Study

**DOI:** 10.1177/26884844251397931

**Published:** 2025-12-05

**Authors:** Emily Newton-Hoe, Elizabeth Janiak, Sarah Johns, Isabel Fulcher, Katharine O. White, Kathryn E. Fay

**Affiliations:** 1 Harvard T.H. Chan School of Public Health, Boston, Massachusetts, USA.; 2 Department of Obstetrics, Gynecology, and Reproductive Biology, Brigham & Women’s Hospital, Boston, Massachusetts, USA.; 3 Delfina Care, San Jose, California, USA.; 4 Department of Obstetrics and Gynecology, Boston Medical Center, Boston, Massachusetts, USA.; 5 Boston University Chobanian & Avedisian School of Medicine, Boston, Massachusetts, USA.; 6 Harvard Medical School, Boston, Massachusetts, USA.

**Keywords:** reproductive coercion, gender-based violence, contraception, pregnancy, health disparities

## Abstract

**Background:**

Reproductive coercion (RC) is a form of intimate partner violence involving behaviors aimed at undermining an individual’s reproductive choices and autonomy. Despite pregnancy representing an important sequela of RC, a time of potential unique risks, and an opportunity for increased health care engagement, limited data exist on the frequency and risk factors of RC among currently pregnant individuals. Thus, we sought to explore the correlates, frequency, and predictors of past RC in a cohort of pregnant people.

**Methods:**

We fielded a cross-sectional survey between February 2022 and September 2023 to pregnant individuals ages 18–49 receiving prenatal care in two tertiary care academic medical centers in Boston, Massachusetts, United States (*N* = 1340).

**Results:**

A total of 76 respondents (7.3%) experienced RC within the past 3 years. The most common form of RC was a partner stopping an individual from using their desired contraceptive method (*N* = 33, 3.2%). Among other differences, people reporting past RC were more likely to have a disability (15.1% vs. 7.5%, *p* = 0.02), and to be in fair or poor health as opposed to good or excellent health (14.5% vs. 7.2%, *p* = 0.03), compared with those not reporting past RC. In a multivariable model adjusting for health status and disability, people reporting past RC had significantly higher odds of describing their current pregnancy as mistimed or unwanted (adjusted odds ratio, 4.30; 95% CI: 2.34, 8.57), compared with those not reporting past RC.

**Conclusions:**

Past RC is prevalent among pregnant individuals in our sample. Disparities across demographic, medical, and social factors highlight opportunities for interventions to prevent recurrent RC post-pregnancy.

## Introduction

Coercive and abusive control of sexual and reproductive health occurs in several contexts in the United States, constituting a dark history of state legislation and clinician overreach from the colonial era to the present.^1,2^ On an interpersonal level, reproductive coercion (RC) is a prevalent form of intimate partner violence (IPV) that affects sexual and reproductive health and may be addressed with pragmatic clinical interventions.^3–5^ Research suggests that the prevalence of RC perpetrated by male intimate partners against female intimate partners in the United States is between 8% and 30%, depending on factors such as the timespan of RC measurement and the age and geographic context of the study sample.^6^ While most of the RC literature in the United States focuses on male perpetration against female intimate partners, RC can occur outside of heterosexual encounters^7,8^ and be perpetrated by nonintimate partners such as family members.^9,10^

RC involves behaviors that interfere with an individual’s reproductive choices and autonomy.^11^ This includes pregnancy pressure (coercion for a partner to become pregnant or prevent pregnancy), contraceptive sabotage (partner interference with contraception), and control of pregnancy outcomes (threats or violence related to pregnancy continuation or termination).^11,12^ Like IPV at large, RC tactics are primarily considered as means of gaining or maintaining power and control beyond achieving a specific reproductive outcome,^4^ although scholarship on perceived perpetrator motivation is still emerging.^13^ Thus, while RC as a form of reproductive control is not bound by biological sex, a central feature of the phenomenon is the power obtained through pro- and anticonception behaviors directed toward pregnancy-capable people.

As such, RC is associated with unintended pregnancy, along with a myriad of other distinct health outcomes, such as increased diagnosis of sexually transmitted infection, decreased sexual self-efficacy, decreased use of contraception, and poor mental health outcomes.^6–8,11,12,14–17^ Furthermore, RC frequently co-occurs with other forms of physical and sexual violence and may increase the risk of more severe violence and polyvictimization.^18–20^ People experiencing interlocking and overlapping effects of racism, ableism, heterosexism, and other drivers of social inequality face the unjust burden of these negative health effects; racialized groups,^15,21,22^ people with disabilities,^23,24^ and sexually minoritized populations^7,8^ are disproportionately impacted by RC and its consequences. These disparities across axes of inequality may be even greater than the data suggest; for example, RC may be underreported in people with disabilities due to a lack of screening shaped by ableist beliefs that disabled people are not sexually active.^25^

Limited data suggest that identification of RC among affected partners transitioning from pregnancy to parenthood is both contextualizing and predictive, meaning these are individuals with increased likelihood of prior IPV, increased risk of ongoing RC, and elevated stressors that may impact their parenting and connection with their child(ren).^26,27^ Pregnancy is also complicated by the well-established link between IPV and poor birth outcomes, shown also among those reporting RC and exacerbated by disparities in access to care and support services.^28–30^ Thus, pregnancy represents an important sequela of RC, a time of potential unique risks, and an opportunity for increased health care engagement, particularly among structurally oppressed populations.

While there is evidence of RC among patients receiving care in family planning centers^11,15^ and obstetrics and gynecology clinics,^18^ as well as among certain sub-groups like people with disabilities,^23,24^ college students,^31^ and previously pregnant individuals,^30^ limited data exist on past experiences of RC among currently pregnant people in the United States. Thus, understanding the frequency and correlates of past RC in a pregnant population is critically needed to prevent recurrent RC in the perinatal period. This article aims to fill this gap in the literature by examining male-perpetrated RC against female intimate partners in the 3 years leading up to pregnancy, particularly in the context of demographic, medical, and social characteristics.

## Materials and Methods

### Sample

We used baseline data from a prospective longitudinal cohort study of pregnant people receiving prenatal care at two tertiary care academic medical centers located in the northeastern United States. One medical center has approximately 2400 births per year^32^ and a 29% Medicaid population^33^ and the other has approximately 6200 births per year^34^ and a 9% Medicaid population.^33^ We restricted participation to people who spoke English or Spanish, were at least 18 years of age, and were at least 24 weeks pregnant and before birth. We focused on patients in later gestation because these pregnancies were more likely to progress to term, thus providing more stable study conditions and enabling longitudinal follow-up in the parent study, although the present analysis uses only baseline data.

We programmed a survey in REDCap with an estimated burden of 15 minutes. The survey was fielded from February 2022 to September 2023. Research assistants screened patients for eligibility and obtained informed consent. Patients completed the survey on their own, either in the clinic or at home, according to their preference. We did not collect data on whether participant characteristics differed by survey setting. Those who completed the survey received $15 remuneration. This study was approved by the Mass General Brigham Institutional Review Board (2021P000762).

### Measures

To address potential bias, survey items were drawn from widely used and validated survey instruments, with adaptations consistent with prior studies. These surveys include the American Community Survey^35^; Pregnancy Risk Assessment Monitoring System^36^; Patient-Reported Outcomes Measurement Information System^37^; Work, Family & Health Study;^38^ and National Longitudinal Study of Adolescent Health.^39^ Survey items assessed demographic characteristics, medical factors, and dimensions of social and decision-making support.

We transformed 16 variables for modeling purposes (Supplementary Table S1). We created categorical variables for age (18–29, ≥30); disability status (no disability, at least one disability); country of birth (outside of United States, within the United States); education (no college, some college, or more); marital status (not married/partnered, married/partnered); prior births, abortions, miscarriages, or ectopic pregnancies (0, ≥1); health status (less than good health, good health, or better); social support (no social support, has social support); normative beliefs about contraceptive use (partner disapproves, is neutral, approves, not applicable); and contraceptive self-efficacy (somewhat/not at all sure, sure/very sure). Race/ethnicity was categorized as Hispanic, non-Hispanic white, non-Hispanic Black, non-Hispanic Asian, or non-Hispanic Multiracial/other. Participants who identified as Hispanic, Latino, or Spanish origin were classified as Hispanic regardless of reported race; non-Hispanic participants were categorized into mutually exclusive racial groups, with those reporting more than one race classified as non-Hispanic Multiracial/other. We used the median household income in the Boston metropolitan statistical area ($100,750) to classify income levels as low (<$54,999; <55% of $100,750), middle ($55,000-$99,999; ≥55% to <100% of $100,750), or high ($100,000+; ≥100% of $100,750).^40^ We measured reproductive decision making using the *Decision-Making Subscale* of the *Reproductive Autonomy Scale.*^41^ This subscale consists of four items that assess who has the most say in (1) contraceptive method choice, (2) contraceptive use, (3) timing of childbearing, and (4) resolution of an unintended pregnancy. Response options were: my partner (score = 1), both me and my partner (score = 2), or me (score = 3). Item scores were averaged to produce an index score (range: 1–3), with higher values indicating greater autonomy in reproductive decision making.^41,42^ Pregnancy intention was measured with a single item asking how participants felt about becoming pregnant just before the current pregnancy (wanted later, sooner, then, or not at any time)^36^ and was analyzed in its original form.

**FIG. 1.**
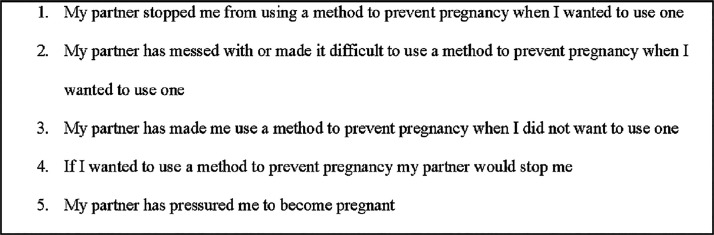
Reproductive coercion questions.

We measured the primary outcome of RC using the *Freedom from Coercion Subscale* from the *Reproductive Autonomy Scale.*^41^ It was asked of people who reported having a sexual partner with a penis in the past 3 years (the preconception period for the current pregnancy). People were instructed to answer in terms of their main partner or a recent sexual partner. Respondents answered five questions on a 1-to-4-point Likert scale from “strongly disagree” to “strongly agree” (Fig. 1). In accordance with the literature, we treated RC as binary with a single positive response indicating RC (*i.e.,* answering “agree” or “strongly agree” to at least one question).^18,43–45^ No data were collected on whether respondent’s current pregnancy was conceived with the person they were thinking of when they answered the questions.

### Analysis

This analysis is limited to eligible pregnant patients who completed the baseline survey. We conducted three analyses with these data. First, we calculated the frequency of past RC by demographic characteristics, medical factors, and social/decision-making support. We tested for potential differences in past RC using chi-square tests or Fisher’s exact tests. Second, we descriptively assessed the types of RC experienced. Third, we conducted a multivariable logistic regression with past RC as our outcome and disability status, physical health status, and pregnancy intention as our predictors. We did not adjust for additional covariates, as the model was designed to evaluate the independent associations of these health-related factors with RC. Statistical significance was set at alpha </= 0.05. Data were cleaned and analyzed using R version 4.3.2.

## Results

### Sample characteristics

**FIG. 2.**
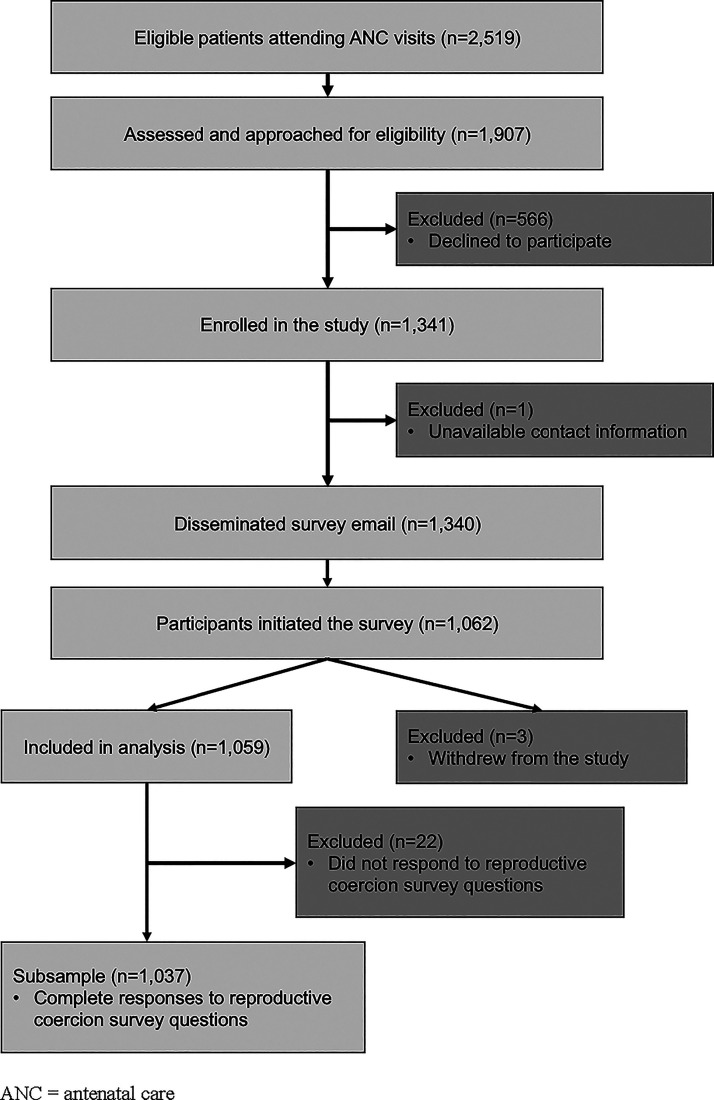
Participant flow diagram.

We distributed 1340 surveys, of which 1062 were opened and begun. After excluding 3 surveys from respondents who later requested their data be removed, we retained 1059 records. Of these, 22 respondents did not respond to RC survey questions, resulting in an analytic sample of 1037 records (Fig. 2).

Respondents reported diverse sociodemographic characteristics in terms of racial/ethnic identity, country of origin, and income level (Table 1). Most were college educated (79.8%), married or partnered (80.5%), age 30 or older (69.9%), and had no disabilities (92.0%). About half wanted to be pregnant at the time they were pregnant (48.0%) and most self-reported being in good or excellent health (92.3%). Nearly all respondents had social support (97.8%) and high contraceptive self-efficacy (88.7%).

**Table 1.** Demographic, Medical, and Social Correlates of Experiences of Reproductive Coercion in the Last 3 Years (*N* = 1037)

		Reproductive coercion		
	Overall *N* (%)	No past RC *N* (%)	Yes past RC *N* (%)	*p* value
Total sample	**1037 obs (100.0%)**	**961 obs (92.7%)**	**76 obs (7.3%)**	—
Demographic characteristics				
Age				0.981
18–29	307 (30.1%)	286 (30.1%)	21 (30.0%)	
30+	712 (69.9%)	663 (69.9%)	49 (70.0%)	
Missing	18	12	6	
Disability status				**0.021**
No disability	929 (92.0%)	867 (92.5%)	62 (84.9%)	
At least one disability	81 (8.0%)	70 (7.5%)	11 (15.1%)	
Missing	27	24	3	
Race/Ethnicity				**<0.001**
Hispanic, Latino, or Spanish origin	210 (21.2%)	189 (20.4%)	21 (31.8%)	
Non-Hispanic white	397 (40.1%)	390 (42.2%)	7 (10.6%)	
Non-Hispanic Black	269 (27.1%)	240 (25.9%)	29 (43.9%)	
Non-Hispanic Asian	72 (7.3%)	65 (7.0%)	7 (10.6%)	
Non-Hispanic Multiracial or other	43 (4.3%)	41 (4.4%)	2 (3.0%)	
Missing	46	36	10	
Country of birth				**<0.001**
Outside of United States	312 (31.2%)	278 (29.9%)	34 (49.3%)	
United States	687 (68.8%)	652 (70.1%)	35 (50.7%)	
Missing	38	31	7	
Education				**0.002**
No college	209 (20.2%)	183 (19.0%)	26 (34.2%)	
Some college or more	828 (79.8%)	778 (81.0%)	50 (65.8%)	
Income				**<0.001**
Low income (<$54,999)	349 (36.2%)	305 (34.1%)	44 (62.9%)	
Middle income ($55,000–$99,999)	114 (11.8%)	108 (12.1%)	6 (8.6%)	
High income (>$100,000)	501 (52.0%)	481 (53.8%)	20 (28.6%)	
Missing	73	67	6	
Marital status				**<0.001**
Not married/partnered	201 (19.5%)	168 (17.6%)	33 (43.4%)	
Currently married/partnered	832 (80.5%)	789 (82.4%)	43 (56.6%)	
Missing	4	4	0	
Medical factors				
Pregnancy intentions				**<0.001**
Did not want to be pregnant then or any time in the future	65 (6.3%)	56 (5.9%)	9 (12.0%)	
Wanted to be pregnant later	215 (21.0%)	179 (18.8%)	36 (48.0%)	
Wanted to be pregnant sooner	254 (24.8%)	236 (24.8%)	18 (24.0%)	
Wanted to be pregnant then	492 (48.0%)	480 (50.5%)	12 (16.0%)	
Missing	11	10	1	
Prior births				0.089
0	458 (48.6%)	433 (49.4%)	25 (38.5%)	
>=1	484 (51.4%)	444 (50.6%)	40 (61.5%)	
Missing	95	84	11	
Prior abortions				**<0.001**
0	659 (75.3%)	623 (76.7%)	36 (57.1%)	
>=1	216 (24.7%)	189 (23.3%)	27 (42.9%)	
Missing	162	149	13	
Prior miscarriages				0.758
0	595 (66.0%)	557 (65.8%)	38 (67.9%)	
>=1	307 (34.0%)	289 (34.2%)	18 (32.1%)	
Missing	135	115	20	
Prior ectopic pregnancies				0.054
0	773 (94.5%)	728 (94.9%)	45 (88.2%)	
>=1	45 (5.5%)	39 (5.1%)	6 (11.8%)	
Missing	219	194	25	
Health status				**0.029**
Less than good health	77 (7.7%)	67 (7.2%)	10 (14.5%)	
Good health or better	921 (92.3%)	862 (92.8%)	59 (85.5%)	
Missing	39	32	7	
Social/decision-making support				
Social support				**0.022**
No social support	23 (2.2%)	18 (1.9%)	5 (6.6%)	
Has social support	1,013 (97.8%)	942 (98.1%)	71 (93.4%)	
Missing	1	1	0	
Normative beliefs about contraceptive use				**<0.001**
Partner disapproves of contraceptive use	34 (3.3%)	26 (2.7%)	8 (10.7%)	
Partner is neutral towards contraceptive use	287 (28.1%)	266 (28.1%)	21 (28.0%)	
Partner approves of contraceptive use	656 (64.3%)	619 (65.4%)	37 (49.3%)	
Not applicable	44 (4.3%)	35 (3.7%)	9 (12.0%)	
Missing	16	15	1	
Reproductive decision making	2.44 (0.37)	2.44 (0.36)	2.44 (0.43)	.970
Missing	27	23	4	
Self-efficacy to use contraception				**<0.001**
Somewhat/not at all sure	115 (11.3%)	94 (10.0%)	21 (28.0%)	
Sure/very sure	902 (88.7%)	848 (90.0%)	54 (72.0%)	
Missing	20	19	1	

Bold values indicate statistically significant *p* value (<0.05).

Data for reproductive decision-making are presented as mean (standard deviation). All other data are presented as *n* (%).

### Analysis 1: Correlates of past RC (Table 1)

Six demographic characteristics were significantly associated with past RC. Pregnant people who had experienced RC in the last three years were significantly more likely to report at least one form of disability (15.1% vs. 7.5%, *p* = 0.021), to have been born outside the United States (49.3% vs. 29.9%, *p* < 0.001), and to have no college education (34.2% vs. 19.0%, *p* = 0.002), compared with those who had not experienced RC in this period. People with past experiences of RC were significantly less likely to self-identify as non-Hispanic white (10.6% vs. 42.2%, *p* < 0.001), to have a high income (28.6% vs. 53.8%, *p* < 0.001), or to be married or partnered (56.6% vs. 82.4%, *p* < 0.001), compared with those without past experiences of RC.

In terms of medical factors, people who had experienced RC in the last 3 years were significantly less likely to have wanted to be pregnant at the time of their pregnancy (16.0% vs. 50.5%, *p* < 0.001), and significantly more likely to report at least one prior abortion (42.9% vs. 23.3%, *p* < 0.001) or to self-report being in poor or fair health (14.5% vs. 7.2%, *p* = 0.029), compared with those who had not experienced RC in this period.

In terms of social and decision-making support, people who had experienced RC in the last 3 years were significantly more likely to report limited social support (6.6% vs. 1.9%, *p* = 0.022), partners who disapprove of contraceptive use (10.7% vs. 2.7%, *p* < 0.001), and low self-efficacy to use contraception as indicated over the next year (28.0% vs. 10.0%, *p* < 0.001), compared with those who had not experienced RC in this period.

### Analysis 2: Frequency of past RC (Table 2)

A total of 76 people (7.3%) reported experiencing at least one form of RC in the past 3 years (Table 2). The most common form of past RC was a partner stopping an individual from using their desired contraceptive method (3.2%). The least common form of past RC was a partner messing with or making it difficult for an individual to use their desired contraceptive method to prevent pregnancy (1.9%).

**Table 2.** Prevalence of Reproductive Coercion in the Last 3 Years (*N* = 76)

	*N* (%)
Any reproductive coercion	**76 (7.3%)**
My partner has stopped me from using a method to prevent pregnancy when I wanted to use one	33 (3.2%)
If I wanted to use a method to prevent pregnancy my partner would stop me	30 (2.9%)
My partner has pressured me to become pregnant	29 (2.8%)
My partner has made me use a method to prevent pregnancy when I did not want to use one	23 (2.2%)
My partner has messed with or made it difficult to use a method to prevent pregnancy when I wanted to use one	20 (1.9%)

Bold values indicate statistically significant *p* value (<0.05).

### Analysis 3: Predictors of past RC (Table 3)

Controlling for disability and physical health status, pregnancy intention was a strong predictor of past experiences of RC, with pregnant people who reported a mistimed or unwanted pregnancy having over four times the odds of past RC compared with pregnant people who wanted to pregnant at that time (95% CI: 2.34, 8.57; *p* < 0.001).

**Table 3.** Odds of Reproductive Coercion in the Last Three Years according to Self-Reported Health Status (*N* = 967)

	AOR	95% CI	*p* value
Disability status			0.116
No disability (ref)	1.00	—	
At least one disability	1.85	0.82, 3.85	
Physical health status			0.253
Good health or better (ref)	1.00	—	
Less than good health	0.64	0.31, 1.45	
Pregnancy intentions			**<0.001**
Wanted to be pregnant then (ref)	1.00	—	
Mistimed or unwanted	4.30	2.34, 8.57	

Bold values indicate statistically significant *p* value (<0.05).

Disability status model controls for physical health status and pregnancy intentions. Physical health status model controls for disability status and pregnancy intentions. Pregnancy intention model controls for disability status and physical health status. No models are adjusted for additional covariates, as each is designed to evaluate the independent association of these health-related factors with reproductive coercion.

AOR, adjusted odds ratio; CI, confidence interval.

## Discussion

Our study found that 7.3% of the sample experienced RC. The frequency of past RC within this sample is slightly lower than estimates in the general U.S. population^21^ and substantially lower than estimates among recently pregnant individuals^30^ and people seeking pregnancy counseling support.^46^ While differences in estimates may be due to factors like the restricted timespan of RC measurement, inconsistencies in how RC was defined, variations in care settings, and cultural factors that differ across local, state, and country contexts, this research nevertheless fills an important gap in understanding past experiences of RC among currently pregnant people.

Differences in the frequency of RC in this sample across demographic, medical, and social factors were also observed. We found higher frequency of past RC among structurally marginalized populations; people with racialized identities, low educational attainment, economic disadvantage, or some form of disability had a significantly higher frequency of past RC compared with their counterparts. In terms of social and environmental factors, we found that people with past experiences of RC reported lower contraceptive self-efficacy, as measured by their confidence in being able to use contraception as indicated over the next year. While our survey did not assess the reasons for this lower confidence, it may signal heightened vulnerability to partner interference in future reproductive decisions and suggest risk of future RC. Indeed, people who experienced RC in the past 3 years also had a significantly higher odds of describing their current pregnancy as mistimed or unwanted compared with those not reporting RC during this period.

While these disparities in RC frequency are consistent with research conducted in the general population of the United States,^21^ our study population of pregnant people further reinforce the health equity implications of addressing RC for pregnancy capable people and their child(ren). Because IPV including RC is a risk factor for adverse maternal and neonatal health outcomes, such as maternal homicide and suicide, low birth weight, preterm birth, and neonatal death,^28–30,47,48^ past experiences of RC exacerbate the existing disparities in maternal and infant health. Thus, addressing disparities in the perinatal setting includes addressing violence, including the interlocking oppressions of racism, classism, and ableism, among others. The frequency of RC in our sample is on par with that of gestational diabetes^49^ and antenatal depression^50^—conditions that are integrated into essential aspects of prenatal care. Situating RC alongside these conditions underscores its relevance to maternal morbidity and mortality, given its potential role in contributing to preventable maternal complications and deaths through pathways such as undesired pregnancy, delayed or foregone care, and constrained reproductive choices. Clinician awareness and universal education about RC during the perinatal period are therefore critical for optimization of obstetrical care more broadly, including recognizing people at greater risk for poor birth outcomes and other forms of violence. Given that about a quarter of people are not screened for IPV during pregnancy,^51^ and when screening does occur, it only takes place during the first prenatal appointment,^52^ implementation of a universal education and screening program may require several provider-, practice-, and policy-level changes.

At the provider level, training may be needed to help clinicians understand RC and its associated health risks, as well as how their own unconscious biases may influence their perceptions of who is (and is not) at risk for RC. Recognizing that RC may cause or be a source of trauma, clinicians can use trauma-informed and survivor-centered approaches, such as Trauma-Informed Personalized Scripts or the evidence-based Confidentiality, Universal Education, Empowerment, and Support (CUES) intervention, which emphasize provision of patient education and resources, even if in the absence of disclosure.^53–55^ Culturally adapted approaches are also needed, such as recognizing the social factors that influence power imbalances across diverse intimate relationships.^15,22,56^ At the practice level, changes may include workflow enhancements that facilitate screening and education in private and at multiple visits.^1^ They may also include updates to the electronic health record to protect sensitive notes on RC from being visible perpetrators, which is especially relevant for people with disabilities who may experience violence from caregivers who have access to their medical record.^57^ Incorporating community resource referrals into the practice workflow is also important, particularly to culturally concordant organizations designed to address the unique needs of diverse groups and moderate the effects of violence.^58^ At the policy level, improvements like altering the reimbursement schema to value time related to screening may be necessary to actualize universal RC screening support.

Even in the absence of universal education and screening for RC, our findings regarding the social and environmental determinants of RC made clear the importance of clinicians providing trauma-informed prenatal care and contraceptive counseling to increase pregnant people’s contraceptive self-efficacy; this may include discussing discreet contraceptive methods less vulnerable to partner influence like intrauterine devices (IUDs), implants, and injectables.^1,4^ Given people experiencing past RC were most likely to have a partner who disapproved of contraceptive use, discussion of how contraceptive implants and IUDs can be placed or other methods can be delivered to the bedside during hospital admission may be attractive to people who are impacted by their coercive partner after birth or at increased risk of recurrent of RC. While potentially reducing pregnancy coercion and eliminating follow-up visits for contraception, people experiencing RC will likely have varied preferences for contraceptive methods beyond their discreet quality.^59,60^ Clinicians should take care in providing contraceptive counseling, avoiding coercion toward contraceptive implants and IUDs as the sole options for people experiencing RC.

Finally, like other studies that have linked unintended pregnancy with RC,^11,12,19,23^ our finding that people who experienced RC in the past 3 years had a significantly higher odds of describing their current pregnancy as mistimed or unwanted compared with those not reporting past RC reinforces the significant impact RC can have on an individual’s reproductive autonomy. We note, however, that the construct of pregnancy intention has been critiqued for oversimplifying complex and dynamic feelings about pregnancy, as well as for its inherent racism and classism,^61^ and our findings should be interpreted with this limitation in mind. In addition, our study may underestimate this association since we excluded those whose pregnancy ended before 24 weeks through induced or spontaneous abortion. It is also unclear if respondents pressured by their partners into pregnancy or prevented from using contraception wanted an abortion but could not access one due to their partners’ actions. While all respondents receive care in Massachusetts, a state protective of abortion access, the overturning of *Roe v. Wade* increases the burdens and barriers associated with obtaining abortions in other states. In many cases, the inability to receive a wanted abortion may make it harder to leave the abusive relationship and lead to sustained coercion and violence for the survivor and possibly their child(ren).^62^ Because abortion access plays a role in reducing IPV,^62^ further research on the impacts of *Roe v. Wade* on RC, violence, mortality, and health disparities will be needed in years to come.

A few limitations to this study should be noted. First, our data are self-reported, potentially resulting in underreporting of RC since people may fear retribution if they acknowledge their partner’s coercive behaviors.^11^ Underreporting may also be because due to the narrow scope of RC tactics queried, including those potentially less applicable to a pregnancy cohort continuing their pregnancies. Second, we dichotomized RC in accordance with the literature,^18,43–45^ classifying any affirmative response as RC and thereby limiting distinctions across experiences. Although the *Freedom from Coercion* subscale has strong validity evidence,^41^ has been applied in other studies to assess RC,^63^ and captures behaviors such as contraceptive nonuse or interfering with method access, it does not include more severe tactics such as threats or physical violence, potentially underestimating the intensity and diversity of RC experiences. As a result, the measure may be sensitive to more common forms of RC while missing less frequent but more severe tactics, and its specificity may exclude subtler or more indirect forms of coercion. In addition, it captures whether RC has occurred but not the duration or severity of experiences, limiting our ability to distinguish between isolated events and ongoing RC. Third, we only capture RC as defined in the survey (“a person with a penis” to a “person with a vagina”), despite evidence that RC occurs outside of heterosexual encounters.^7,8^ Fourth, since our sample is limited to pregnant people receiving care in the northeastern United States, a high resourced area, results may not be generalizable to lower resourced communities. While the sample is reasonably diverse in terms of racialized identity and country of origin, the relatively small sample of people who gave a positive response about RC and homogeneity of other sociodemographic characteristics may also limit generalizability. Fifth, our analytic sample excluded individuals whose pregnancies ended before 24 weeks through abortion or miscarriage, which likely led us to underestimate the frequency of RC in our sample given that these individuals are disproportionately likely to have experienced RC. This exclusion also limits the generalizability of our findings to people with ongoing pregnancies and may not capture the full spectrum of RC experiences among all pregnancy-capable individuals. As such, our estimates should be considered conservative.

## Conclusions

RC is a prevalent form of IPV impacting pregnant people. The disparities in past experiences of RC across demographic, medical, and social factors highlight that addressing disparities in the perinatal setting includes addressing violence. This might include providing universal education about and screening for RC, discussing nuanced contraceptive performance needs among interested patients, and facilitating access to abortion providers.

## Authors’ Contributions

E.N.-H. contributed to data curation, formal analysis, and writing—original draft, review, and editing. E.J., I.F., and K.O.W. contributed to the funding acquisition, project administration, and writing—review and editing. S.J. contributed to project administration, validation, and writing—review and editing. K.E.F. and E.J. contributed to the conceptualization, investigation, and methodology. K.E.F. contributed to writing—original draft, supervision, and validation.

## Supplemental Material

sj-docx-1-whr-10.1177_26884844251397931 – Supplemental material for Frequency and Risk Factors of Reproductive Coercion Among Pregnant Individuals: A Cross-Sectional Study Supplemental material, sj-docx-1-whr-10.1177_26884844251397931 for Frequency and Risk Factors of Reproductive Coercion Among Pregnant Individuals: A Cross-Sectional Study by Emily Newton-Hoe, Elizabeth Janiak, Sarah Johns, Isabel Fulcher, Katharine O. White, and Kathryn E. Fay

## Abbreviations

CUES: Confidentiality, Universal Education, Empowerment, and Support
IPV: intimate partner violence
IUD: intrauterine device
RC: reproductive coercion
