# Supplementary material for: Frequency and Risk Factors of Reproductive Coercion Among Pregnant Individuals: A Cross-Sectional Study
Source: Womens Health Rep (New Rochelle). 2025 Dec 5;6(1):1252–62. doi: 10.1177/26884844251397931 (PMC13185944; doi:10.1177/26884844251397931)
Supplement: sj-docx-1-whr-10.1177_26884844251397931 – Supplemental material for Frequency and Risk Factors of Reproductive Coercion Among Pregnant Individuals: A Cross-Sectional Study [file sj-docx-1-whr-10.1177_26884844251397931.docx]

**Supplemental Table 1. Survey question wording for key variables and categories used in analysis**

| **Variable** | **Survey question** | **Categories used in analysis** |
| --- | --- | --- |
| **Demographic characteristics** | | |
| Age^a^ | What is your age in years? ___________ (free response) | - 18-29 - 30+ |
| Disability status^a^ | Are you deaf, or do you have serious difficulty hearing?   - Yes - No   Are you blind, or do you have serious difficulty seeing, even when wearing glasses?   - Yes - No   Because of a physical, mental, or emotional condition, do you have serious difficulty concentrating, remembering, or making decisions?   - Yes - No   Do you have serious difficulty walking or climbing stairs?   - Yes - No   Do you have difficulty dressing or bathing?   - Yes - No   Because of a physical, mental, or emotional condition, do you have difficulty doing errands alone such as visiting a doctor’s office or shopping?   - Yes - No | - No disability - At least one disability |
| Race^a^ | What is your race? *Check all that apply*   - White - Black or African American - American Indian or Alaska Native - Asian - Native Hawaiian - Other Pacific Islander - Other race | - Hispanic, Latino, or Spanish origin - Non-Hispanic white - Non-Hispanic Black - Non-Hispanic Asian - Non-Hispanic Multiracial or other |
| Ethnicity^a^ | Are you Hispanic, Latino, or Spanish origin? *Check all that apply*   - No, not of Hispanic, Latino, or Spanish origin - Yes, Mexican, Mexican American, Chicano - Yes, Puerto Rican - Yes, Cuban - Yes, other Hispanic, Latino, or Spanish origin |  |
| Country of birth^a^ | In what country were you born? ___________ (free response) | - Outside of USA - USA |
| Education^a^ | What is the highest degree or level of schooling you have completed?   - Less than 12 years of school (no high school diploma or equivalent) - High school diploma - GED, HiSET, or TASC credential - Some college credit (no degree or in progress) - Associate’s degree (for example: AA, AS) - Bachelor’s degree (for example: BA, BS) - Graduate degree (master’s, professional, or doctorate) | - No college - Some college or more |
| Income | Now we are going to ask you about your yearly household income from all sources. Please think about all income from jobs, alimony, et cetera, from everyone who lives in your house.   - Less than $10,000 - $10,000 – $14,999 - $15,000 – $44,999 - $45,000 – $54,999 - $55,000 – $99,999 - $100,000+ | - Low income (<$54,999) - Middle income ($55,000-$99,999) - High income (>$100,000) |
| Marital status^a^ | Are you currently married or living with a partner?   - Yes - No | - Not married/ partnered - Currently married/ partnered |
| **Medical factors** | | |
| Pregnancy intentions^b^ | Think back to *just before* you got pregnant with this pregnancy, how did you feel about becoming pregnant? (choose only one)   - I wanted to be pregnant later - I wanted to be pregnant sooner - I wanted to be pregnant then - I didn’t want to be pregnant then or at any time in the future | Same as original variable |
| Prior births | Please indicate how many of the following you have had:  Prior births: __________ | - 0 - >=1 |
| Prior abortions | Please indicate how many of the following you have had:  Prior abortions (pregnancy terminations): __________ | - 0 - >=1 |
| Prior miscarriages | Please indicate how many of the following you have had:  Prior miscarriages: __________ | - 0 - >=1 |
| Prior ectopic pregnancies | Please indicate how many of the following you have had:  Prior ectopic (tubal) pregnancies: __________ | - 0 - >=1 |
| Health status^c^ | In general, how would you rate your physical health?   - Excellent - Very good - Good - Fair - Poor | - Less than good health - Good health or better |
| **Social/decision-making support** | | |
| Social support^d^ | Can you count on a friend or relative to provide you with emotional support?   - Yes – always - Yes – sometimes - No – there isn’t anyone like that | - No social support - Has social support |
| Normative beliefs about contraceptive use^e^ | How would your partner feel about you using birth control after this pregnancy?   - Strongly disapprove - Disapprove - Neither approve nor disapprove - Approve - Strongly approve - This does not apply to me; I do not have a partner | - Partner disapproves of contraceptive use - Partner is neutral towards contraceptive use - Partner approves of contraceptive use - Not applicable |
| Reproductive decision making^f^ | Who has the MOST say about which method you would use to prevent pregnancy?   - My partner - Both me and my partner - Me   Who has the MOST say about when you have a baby in your life?   - My partner - Both me and my partner - Me   Who has the MOST say about whether you use a method to prevent pregnancy?   - My partner - Both me and my partner - Me   If you become pregnant but it was unplanned, who would have the MOST say about whether you would raise the child, seek adoptive parents, or have an abortion?   - My partner - Both me and my partner - Me | - Partner makes decisions - Shared decisions |
| Self-efficacy to use contraception^e^ | How sure are you that you could use a method of contraception as indicated over the course of the next year if you were sexually active with a man and wanted to avoid pregnancy?   - Very sure - Sure - Somewhat sure - Not at all sure | - Somewhat/not at all sure - Sure/very sure |
| **Reproductive coercion** | | |
| Reproductive coercion^f^ | My partner has stopped me from using a method to prevent pregnancy when I wanted to use one   - Strongly disagree - Disagree - Agree - Strongly agree   My partner has messed with or made it difficult to use a method to prevent pregnancy when I wanted to use one.   - Strongly disagree - Disagree - Agree - Strongly agree   My partner has made me use a method to prevent pregnancy when I did not want to use one.   - Strongly disagree - Disagree - Agree - Strongly agree   If I wanted to use a method to prevent pregnancy my partner would stop me.   - Strongly disagree - Disagree - Agree - Strongly agree   My partner has pressured me to become pregnant.   - Strongly disagree - Disagree - Agree - Strongly agree | - Yes RC - No RC |

^a^ Survey items adapted from The American Community Survey.^35^

^b^ Survey item from the Pregnancy Risk Assessment Monitoring System.^36^

^c^ Survey item from the Patient-Reported Outcomes Measurement Information System.^37^

^d^ Survey item adapted from the Work, Family & Health Study.^38^

^e^ Survey items adapted from the National Longitudinal Study of Adolescent Health.^39^

^f^ Survey items from the Reproductive Autonomy Scale.^41^
